# Association of plasma ghrelin levels and ghrelin rs4684677 polymorphism with mild cognitive impairment in type 2 diabetic patients

**DOI:** 10.18632/oncotarget.14852

**Published:** 2017-01-27

**Authors:** Rong Huang, Jing Han, Sai Tian, Rongrong Cai, Jie Sun, Yanjue Shen, Shaohua Wang

**Affiliations:** ^1^ Department of Endocrinology, Affiliated ZhongDa Hospital of Southeast University, Nanjing, PR China, 210009; ^2^ Medical School of Southeast University, Nanjing, PR China, 210009

**Keywords:** ghrelin, polymorphism, mild cognitive impairment, type 2 diabetes mellitus

## Abstract

**Background and aims:**

People with insulin resistance and type 2 diabetes mellitus (T2DM) are at increased risks of cognitive impairment. We aimed to investigate the association of plasma ghrelin levels and ghrelin rs4684677 polymorphism with mild cognitive impairment (MCI) in T2DM patients.

**Results:**

In addition to elevated glycosylated hemoglobin (HbA1c), fasting blood glucose (FBG) and homeostasis model assessment of insulin resistance (HOMA-IR), T2DM patients with MCI had decreased plasma ghrelin levels compared with their healthy-cognition subjects (all *p* < 0.05). Further logistic regression analysis showed that ghrelin level was one of independent factors for MCI in T2DM patients (*p* < 0.05). Moreover, partial correlation analysis demonstrated that ghrelin levels were positively associated with the scores of Montreal Cognitive Assessment (*r* = 0.196, *p* = 0.041) and Auditory Verbal Learning Test-delayed recall (*r* = 0.197, *p* = 0.040) after adjustment for HbA1c, FBG and HOMA-IR, wherein the latter represented episodic memory functions. No significant differences were found for the distributions of genotype and allele of ghrelin rs4684677 polymorphism between MCI and control group.

**Materials and methods:**

A total of 218 T2DM patients, with 112 patients who satisfied the MCI diagnostic criteria and 106 who exhibited healthy cognition, were enrolled in this study. Demographic characteristics, clinical variables and cognitive performances were extensively assessed. Plasma ghrelin levels and ghrelin rs4684677 polymorphism were also determined.

**Conclusions:**

Our results suggest that decreased ghrelin levels are associated with MCI, especially with episodic memory dysfunction in T2DM populations.

## INTRODUCTION

Type 2 diabetes mellitus (T2DM) is associated with complications, including retinopathy, nephropathy, cardiovascular and cerebrovascular diseases, as well as peripheral neuropathy. Longitudinal studies reported that patients with T2DM have an increased risk for cognitive deficits, such as Alzheimer's disease (AD), vascular dementia, and mild cognitive impairment (MCI) [1]. Previous researches also indicated that diabetes is related to decrements in several cognitive domains, including processing speed, executive function, learning, and memory [2, 3]. Although many mechanisms were explored, information is lacking regarding the exact mechanisms involved in T2DM-associated cognitive impairments.

Ghrelin, a 28-amino acid gastrointestinal peptide, was first discovered as the endogenous ligand for the growth hormone secretagogue receptor 1a by Kojima and Kangawa in 1999 [4]. Both ghrelin and its receptor are widely expressed in peripheral tissues and multiple regions of the brain, such as the intestine, pancreatic islets, pituitary, hippocampus and arcuate nucleus [5, 6]. Consequently, numerous peripheral and central functions of ghrelin were described; these functions include stimulation of gastric acid secretion, regulation of glucose metabolism, modulation of sleep, stress and anxiety, and regulation of learning and memory [7–11]. Previous studies showed that ghrelin level is lower in individuals showing obesity, insulin resistance, or metabolic syndrome [12–14], and all these conditions were proven to be risk factors for cognitive decline in T2DM subjects [15, 16]. Moreover, an animal study suggested that ghrelin might improve spatial learning and memory performance via a central nervous system mechanism involving insulin signaling [17].

The human ghrelin gene is located in chromosome 3p26-25 and consists of four exons and three introns [18]. The most studied SNPs to date are Arg51Gln, Gln90Leu (rs4684677), and Leu72Met (rs696217). Among these SNPs, 269A > T, a mutation which causes the Gln90Leu change in the prepro-ghrelin, was described to be associated with AD in a Japanese case-control study [19]. Rs4684667 A/T also showed significant association with cognitive impairment in elder Spanish community dwelling individuals [20]. A variety of other studies provided supporting evidence for the role of rs4684677 T/A polymorphism in influencing metabolic syndrome, panic disorder, cancer, and autoimmune thyroid disease risks [21–24].

However, the relationship among plasma ghrelin, ghrelin polymorphism, and cognitive function in T2DM patients is currently unknown. Given the animal research that suggests a link between ghrelin and cognition, as well as inconsistent findings from human studies, we hypothesized that ghrelin, which plays important roles in insulin resistance and metabolic syndrome, may be involved in the early stage of diabetes-associated cognitive decline. Therefore, this study was designed to investigate the potential association between plasma ghrelin levels and different domains of cognitive performances, and to determine whether rs4684677 polymorphism in ghrelin gene is associated with mild cognitive impairment in T2DM subjects.

## RESULTS

### Sociodemographic characteristics, clinical variables and cognitive performances

Sociodemographic characteristics, clinical variables and neuropsychological test scores of the participants are summarized in Table 1. The MCI and control groups were well matched in terms of age, gender, and education levels (*p* > 0.05). Type 2 diabetic patients with MCI had elevated glycosylated hemoglobin (HbA1c), fasting blood glucose (FBG) and homeostasis model assessment of insulin resistance (HOMA-IR) compared with the control group (all *p* < 0.05). The prevalence of diabetic nephropathy (DN) in the MCI group was significantly higher than that in the control group (*p* = 0.035). No significant differences were observed regarding the other characteristics between the two groups (all *p* > 0.05). Moreover, T2DM with MCI patients displayed significantly poorer overall and different domains of cognitive performances than control subjects (all *p* < 0.05).

**Table 1:** Demographic characteristics, clinical variables and cognitive performances

Characteristic	MCI group (*n* = 112)	Non-MCI group (*n* = 106)	*p*-value
**Age (years)**	61.23 ± 7.19	59.61 ± 7.56	0.106 ^a^
**Female,** ***n*** **(%)**	52 (46.43)	37 (34.91)	0.084 ^c^
**Education Levels (years)**	9.00 (9.00–12.00)	10.00 (9.00–12.25)	0.245 ^b^
**Smoking,** ***n*** **(%)**	47 (41.96)	48 (45.28)	0.621 ^c^
**BMI (kg/m^2^)**	25.19 ± 3.68	25.00 ± 3.33	0.702 ^a^
**Hypertension,** ***n*** **(%)**	72 (64.29)	58 (54.72)	0.150 ^c^
**Diabetes duration (years)**	10.00 (5.00–15.00)	8.00 (5.00–12.00)	0.266 ^b^
**HbA1c (%)**	9.54 ± 2.45	8.84 ± 2.28	0.030 ^a^
**FBG**	7.38 (6.20–9.99)	7.04 (5.62–8.46)	0.033 ^b^
**FIN**	18.29 ± 5.77	17.25 ± 5.56	0.177 ^a^
**HOMA-IR**	0.86 (0.70–1.05)	0.73 (0.62–0.85)	< 0.001 ^b^
**Insulin usage,** ***n*** **(%)**	77 (68.75)	61 (57.55)	0.086 ^c^
**Triglyceride(mmol/L)**	1.78 (1.14–2.65)	1.49 (1.14–2.52)	0.335 ^b^
**Total cholesterol (mmol/L)**	4.98 ± 1.58	4.86 ± 1.23	0.536 ^a^
**HDL- cholesterol (mmol/L)**	1.20 ± 0.31	1.19 ± 0.25	0.701 ^a^
**LDL- cholesterol (mmol/L)**	2.93 ± 0.89	2.95 ± 0.87	0.826 ^a^
**CHD,** ***n*** **(%)**	21 (18.75)	12 (10.71)	0.126 ^c^
**DN,** ***n*** **(%)**	16 (14.29)	6 (5.66)	0.035 ^c^
**DR,** ***n*** **(%)**	9 (8.04)	6 (5.66)	0.489 ^c^
**Ghrelin (pg/mL)**	190.55 (121.75–244.43)	222.62 (140.89–346.92)	0.011 ^b^
**Neuropsychological test scores**			
**MoCA**	22.00 (20.00–24.00)	27.00 (26.00–28.00)	< 0.001 ^b^
**DST**	11.00 (9.00–12.00)	12.00 (11.00–14.00)	< 0.001 ^b^
**VFT**	15.53 ± 3.89	16.84 ± 3.83	0.013 ^a^
**CDT**	3.00 (3.00–4.00)	4.00 (3.00–4.00)	0.036 ^b^
**ST**	7.00 (6.00–9.00)	9.00 (7.00–10.00)	< 0.001 ^b^
**TMT-A**	71.00 (57.25–96.75)	59.00 (47.75–71.00)	< 0.001 ^b^
**TMT-B**	182.50 (149.25–239.25)	144.00 (112.25–180.00)	< 0.001 ^b^
**AVLT-immediate recall**	17.00 (13.00–21.00)	19.00 (17.75–23.00)	< 0.001 ^b^
**AVLT-delayed recall**	6.00 (4.00–7.00)	7.00 (6.00–8.00)	< 0.001 ^b^

Data are presented as *n* (%), mean ± SD, or median (interquartile range) as appropriate.

^a^ Student's *t* test for comparison of normally distributed quantitative variables between MCI group and control group.

^b^ Mann-Whitney *U* test for comparison of asymmetrically distributed quantitative variables between MCI group and control group.

^c^ χ^2^ test for comparison of qualitative variables between MCI group and control group.

Abbreviations: MCI, mild cognitive impairment; BMI, body mass index; HbA1c, glycosylated hemoglobin; FBG, fasting blood glucose; FIN, fasting insulin; HOMA-IR, the homeostasis model assessment of insulin resistance; HDL, high-density lipoprotein; LDL, low-density lipoprotein; CHD, coronary artery disease; DN, Diabetic nephropathy; DR, Diabetic retinopathy. MoCA, Montreal Cognitive Assessment; DST, Digit Span Test; VFT, Verbal Fluency Test; CDT, Clock Drawing Test; ST, Similarities test; TMT-A, Trail Making Test-A; TMT-B, Trail Making Test-B; AVLT, Auditory Verbal Learning Test.

### Plasma ghrelin levels in MCI and control subjects

**Figure 1:**
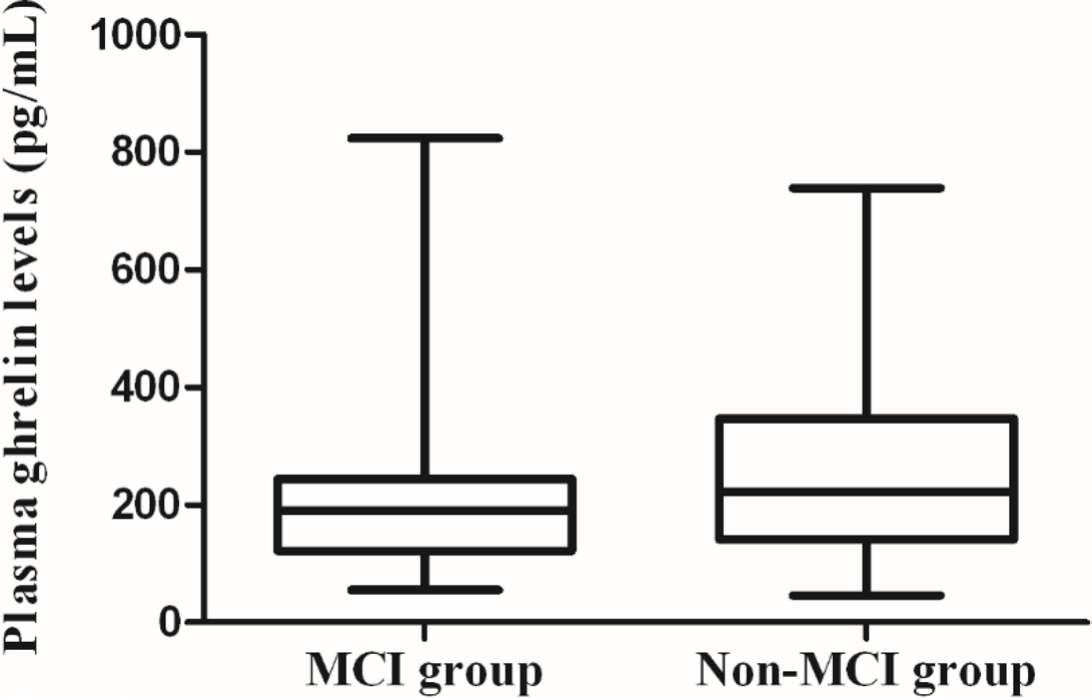
Plasma ghrelin levels in MCI and control subjects Plasma ghrelin levels in MCI patients were significant lower than that of controls (190.55[121.75–244.43] vs 222.62[140.89–346.92] pg/mL, *p* = 0.011).

Plasma ghrelin levels in MCI patients were significantly lower than that of control subjects (190.55[121.75–244.43] vs 222.62[140.89–346.92] pg/mL, *p* = 0.011) (Figure 1). Further logistic regression analysis showed that HOMA-IR, ghrelin levels and DN were independent factors for MCI in T2DM patients (all *p* < 0.05). In addition, plasma ghrelin levels were inversely correlated with body mass index (BMI) (*r* = −0.279, *p* = 0.003), fasting insulin (FIN) (*r* = −0.203, *p* = 0.032), and HOMA-IR (*r* = −0.222, *p* = 0.019) in MCI patients. However, no significant associations were found between ghrelin levels and age, education level, BMI, and clinical variables in normal control subjects (all *p* > 0.05).

### Correlation between plasma ghrelin levels and cognitive performances

**Figure 2:**
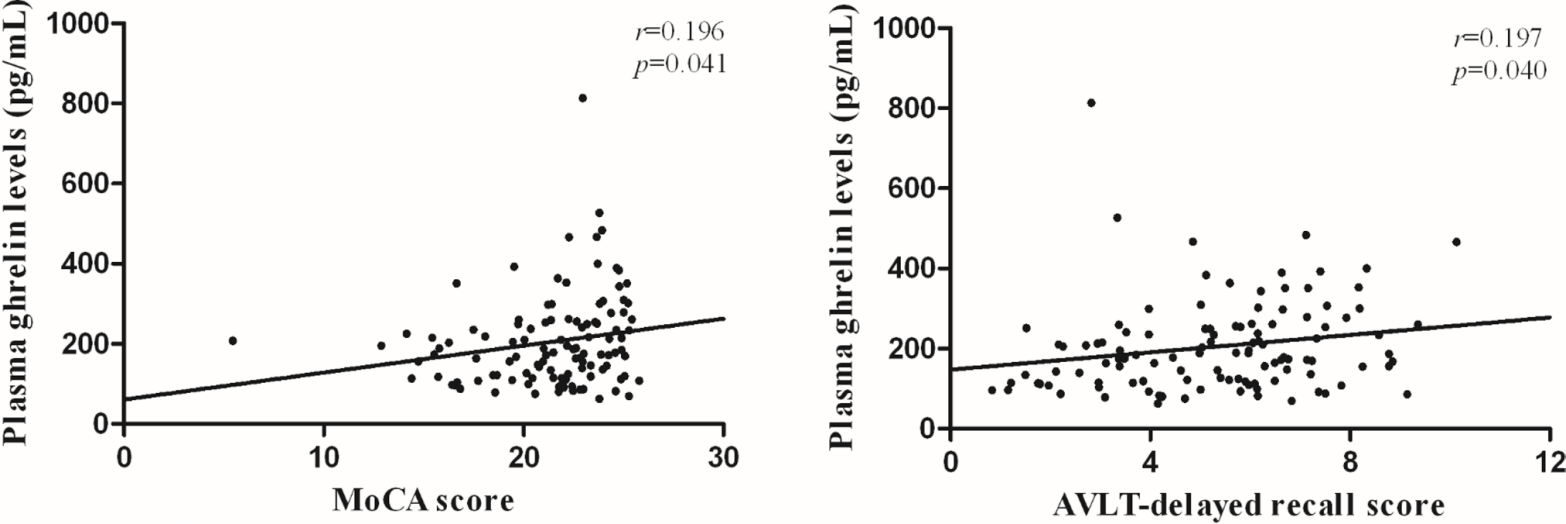
Correlation between plasma ghrelin levels and cognitive performances Partial correlation analysis demonstrated that ghrelin levels were positively associated with the scores of Montreal Cognitive Assessment (MoCA) (*r* = 0.196, *p* = 0.041) and Auditory Verbal Learning Test (AVLT) -delayed recall (*r* = 0.197, *p* = 0.040) in T2DM with MCI patients. Partial *r* and *p* values were obtained after adjustment for HbA1c, FBG and HOMA-IR.

Partial correlation analysis showed that plasma ghrelin levels are not correlated with the overall cognition or any domain of cognitive performances after adjustment for HbA1c, FBG and HOMA-IR in control subjects. However, ghrelin levels were positively associated with the scores of Montreal Cognitive Assessment (MoCA) in MCI patients (*r* = 0.196, *p* = 0.041). We therefore analyzed different domains of cognitive performances, and observed positive association between plasma ghrelin levels and scores of Auditory Verbal Learning Test (AVLT)-delayed recall (*r* = 0.197, *p* = 0.040), which represents delayed episodic memory function (Figure 2).

### Distributions of ghrelin genotype and allele frequencies between groups

Table 2 shows ghrelin genotype and allele frequencies of MCI patients and control subjects. The homozygous (TT) and heterozygous (TA) genotype frequencies of ghrelin rs4684677 were 93.75% and 6.25% in the case group, and 95.28% and 4.72% in the control group, respectively. Distribution of the ghrelin rs4684677 genotypes was consistent with the Hardy–Weinberg equilibrium for both the MCI (χ^2^ = 0.12, df = 1, *p* > 0.05) and control groups (χ^2^ = 0.06, df = 1, *p* > 0.05). No significant differences were observed in the distributions of ghrelin genotypes (χ^2^ = 0.246, df = 1, *p* = 0.620) and allele frequencies (χ^2^ = 0.239, df = 1, *p* = 0.625) between the two groups.

**Table 2:** Distributions of ghrelin genotype and allele frequencies between groups

Group	MCI, *n*(%)	Non-MCI, *n*(%)	OR (95% CI)	*p*-value ^a^
**Overall**	112	106		
**TT**	105 (93.75)	101 (95.28)	1.000	
**TA**	7 (6.25)	5 (4.72)	1.347 (0.414–4.381)	0.620
**T**	217 (96.88)	207 (97.64)	1.000	
**A**	7 (3.12)	5 (2.36)	1.335 (0.417–4.274)	0.625

Data are presented as *n* (%).

^a^χ^2^ test for comparison of genotype and allele frequencies between MCI group and control group.

Abbreviations: MCI, mild cognitive impairment; OR, odds ratio; CI, confidence interval.

### Comparison of plasma ghrelin levels and cognitive performances between genotypic subgroups

Main effects of ghrelin rs4684677 polymorphism on plasma ghrelin levels and cognitive performances in the MCI and control subjects are summarized in Table 3. No significant differences were noted between genotypic subgroups (TT and TA) of ghrelin rs4684677 polymorphism and plasma ghrelin levels in either the MCI or control group (*p* > 0.05). The overall cognition and different domains of cognitive performances were also not significantly different between genotypic subgroups in both groups (*p* > 0.05).

**Table 3:** Comparison of plasma ghrelin levels and cognitive performances between genotypic subgroups

	MCI group			Non-MCI group		
	TT (*n* = 105)	TA (*n* = 7)	*p*-value	TT (*n* = 101)	AT (*n* = 5)	*p*-value
**Ghrelin**	193.16 (122.44–243.85)	161.38 (106.90–267.82)	0.759 ^b^	224.57 (139.44–349.17)	202.35 (137.53–337.62)	0.876 ^b^
**MoCA**	22.00 (20.00–24.00)	20.00 (19.00–22.00)	0.162 ^b^	27.00 (26.00–28.00)	27.00 (26.50–28.00)	0.753 ^b^
**DST**	11.00 (9.00–12.00)	10.00 (10.00–11.00)	0.495 ^b^	12.00 (11.00–14.00)	12.00 (11.50–14.50)	0.576 ^b^
**VFT**	15.53 ± 3.74	15.43 ± 6.13	0.945 ^a^	16.96 ± 3.80	14.40 ± 4.22	0.146 ^a^
**CDT**	4.00 (3.00–4.00)	4.00 (3.00–4.00)	0.558 ^b^	4.00 (3.00–4.00)	3.00 (2.50–4.00)	0.325 ^b^
**ST**	7.00 (5.50–9.00)	8.00 (7.00–9.00)	0.569 ^b^	9.00 (7.50–10.00)	9.00 (7.00–10.50)	0.994 ^b^
**TMT-A**	71.00 (59.00–96.50)	84.00 (53.00–108.00)	0.914 ^b^	60.00 (46.50–71.00)	58.00 (50.00–83.50)	0.754 ^b^
**TMT-B**	193.00 (150.00–240.00)	164.00 (130.00–213.00)	0.315 ^b^	144.00 (111.50–179.50)	155.00 (124.00–196.50)	0.498 ^b^
**AVLT-immediate recall**	17.00 (13.00–21.00)	15.00 (11.00–23.00)	0.718 ^b^	19.00 (17.00–23.00)	19.00 (18.00–20.00)	0.554 ^b^
**AVLT-delayed recall**	6.00 (4.00–7.00)	6.00 (3.00–6.00)	0.706 ^b^	7.00 (6.00–8.50)	7.00 (6.00–7.50)	0.750 ^b^

Data are presented as mean ± SD, or median (interquartile range) as appropriate.

^a^ Student's *t* test for comparison of normally distributed quantitative variables between genotypic subgroups in MCI group and control group.

^b^ Mann-Whitney *U* test for comparison of asymmetrically distributed quantitative variables between genotypic subgroups in MCI group and control group.

Abbreviations: MCI, mild cognitive impairment; MoCA, Montreal Cognitive Assessment; DST, Digit Span Test; VFT, Verbal Fluency Test; CDT, Clock Drawing Test; ST, Similarities test; TMT-A, Trail Making Test-A; TMT-B, Trail Making Test-B; AVLT, Auditory Verbal Learning Test.

## DISCUSSION

The main results of this study were as follows: (1) Compared with T2DM patients with healthy cognition, plasma ghrelin levels were severely decreased in those with MCI. (2) Decreased ghrelin levels were one of the independent factors for MCI in T2DM patients. (3) Plasma ghrelin levels were positively correlated with overall cognitive function, especially with delayed memory index in T2DM subjects with MCI. (4) No association was found between rs4684677 polymorphism and MCI in T2DM population. (5) Genotype did not significantly affect plasma ghrelin levels and different domains of cognitive performances in both MCI patients and healthy-cognition control subjects.

In the current study, we first conducted a correlation study of plasma ghrelin levels and cognitive performances in T2DM patients, and observed reduced ghrelin levels in T2DM subjects with MCI. These findings are consistent with those in animal literature, which suggested the association between elevated ghrelin and improved hippocampal functions [25, 26]. Interestingly, we observed that BMI, FIN, and HOMA-IR were negatively associated with ghrelin, suggesting that decreased ghrelin levels in MCI patients may be related to higher BMI, FIN, and HOMA-IR. Previous study indicated that fasting plasma ghrelin levels were decreased in obese subjects [12]. Moreover, diet-induced obesity could suppress the neuroendocrine ghrelin axis by decreasing total plasma ghrelin levels [27]. One recent cohort study also showed a significant association between plasma ghrelin and HOMA-IR index, which reflects insulin sensitivity [28]. Furthermore, the negative associations between circulating ghrelin and insulin resistance, as well as insulin levels in this study are consistent with those found in a large population-based study [29]. All the aforementioned factors were proven to contribute to cognitive dysfunction in T2DM subjects [15, 30]. Thus, decreased ghrelin may be a pathogenetic factor involved in T2DM-associated cognitive impairment.

We noted that lower plasma ghrelin levels were correlated with poorer overall cognitive performances, especially poorer delayed episodic memory function in T2DM patients with MCI. This result is in contrast to that of a recent study conducted on a sample of non-demented elderly; the particular study indicated that serum ghrelin levels are inversely associated with several cognitive domains, including verbal memory, working memory, and naming [31]. The exact mechanisms for these relationships are not fully understood. One possible reason is that ghrelin exhibits dense receptor expression in the hippocampus, where it has been proved to form learning and memory function in rodents [25]. Ghrelin administration also promotes long term potentiation (LTP) in the hippocampus; LTP is a phenomenon that positively correlates with learning and memory [32]. Moreover, ghrelin can increase spine synapse density in the hippocampal CA1 region, therefore enhancing performances in several types of hippocampal-dependent learning and memory tasks [33]. In addition, ghrelin administration improved the cognitive ability in streptozotocin-induced diabetic rats by improving the expressions of brain-derived neurotrophic factor and cAMP-response element binding and by attenuating hippocampal neuronal apoptosis [34]. Animal experiments also demonstrated that behavioral alterations of db/db mice are associated with increased inflammatory cytokines in the hippocampus [35]. Early treatment with ghrelin can inhibit proinflammatory responses in the hippocampus and prevent cognitive impairment in septic rats [36].

We also compared the distributions of genotype and allele frequencies of ghrelin rs4684677 polymorphism between T2DM patients with MCI and control subjects. No significant association was found between ghrelin rs4684677 polymorphism and MCI in our diabetic subjects. Further analysis did not show any significant relationship between ghrelin rs4684677 genotypes and plasma ghrelin levels, as well as different domains of cognitive performances in either MCI or control group. This result was inconsistent with a Japanese case-control study which showed significant association between rs4684677 polymorphism and AD [19]. Moreover, another study showed that L90G ghrelin gene variant influenced cognitive performances in old dwelling individuals participating in the Mataro´ Ageing Study [20]. These discrepancies might be explained by several factors. The subjects included in our study are Chinese Han and T2DM patients; racial and population differences may exist. In addition, the power of a test was only 7.92% based on the low mutation frequency and small sample size of our study, which might lead to this conflicting result. T2DM-associated cognitive impairment is a polygenic hereditary disorder, in which several genes and gene-environment interactions may play an important role in the pathogenesis of the disease [37].

Our study first investigated the association among plasma ghrelin levels, ghrelin rs4684677 polymorphism, and cognitive performances in T2DM patients. However, several limitations should be noted in this study. First, findings derived from a case-control study cannot shed light on the direction of the relationship, and it is not possible to determine causality. Moreover, although previous studies showed that ghrelin can cross the blood-brain barrier, no direct evidence proves that peripheral ghrelin levels may reflect the existence of the peptide levels of in the brain. In addition, in this study, we measured total plasma ghrelin levels, in which approximately 80%–90% of circulating ghrelin is des-acyl ghrelin; ghrelin was originally supposed to be biologically active only in its acylated form [38]. Further studies should clarify whether des-acyl ghrelin represents a precursor or degradation product of the acylated peptide and its specific roles [39]. Finally, the relatively small sample size and sample composition of this study limited the interpretation of our results to a certain degree. T2DM is also a multifactorial disease, in which differences between groups may strongly influence the conclusion,

In conclusion, plasma ghrelin was reduced in T2DM patients with MCI, thereby providing additional evidence that lower ghrelin levels significantly correlate with T2DM-associated cognitive impairment, especially with poor episodic memory function in T2DM with MCI subjects. However, we failed to detect any association between rs4684677 polymorphism of ghrelin gene and risk of MCI. After combining the results, we hypothesize that decreased ghrelin levels may be a risk factor for cognitive defects in T2DM population. Further prospective studies with a substantial sample size should be conducted to confirm these observed findings.

## MATERIALS AND METHODS

### Subjects

A total of 218 T2DM subjects (129 men and 89 women, age ranging from 45–75 years old, and education level ≥ 6 years) were recruited from the Department of Endocrinology, Affiliated ZhongDa Hospital of Southeast University between August 2013 and August 2015. Out of the 218 subjects, 112 were diabetic patients with MCI and 106 were age, gender, and education level-matched diabetic patients with healthy cognition. All T2DM patients were diagnosed based on the World Health Organization (1999) criteria [40]. Meanwhile, the MCI subjects satisfied the following diagnostic criteria for MCI proposed by the MCI Working Group of the European Consortium on Alzheimer's Disease in 2006: 1) cognitive complaints from the patient or family of the patient; 2) a reported decline in cognitive function relative to that in the past year by the patient or guardian of the patient (Clinical Dementia Rating (CDR) score of 0.5); 3) cognitive disorders as evidenced by a clinical evaluation (impairment in memory or other cognitive domains); 4) absence of major repercussions in activities of daily living; and 5) absence of dementia (based on the Diagnostic and Statistical Manual of Mental Disorders-IV criteria) [41]. Individuals with diabetic ketoacidosis, hyperosmolarnonketotic diabetic coma, severe hypoglycemia, and acute cardiovascular or cerebrovascular accident were excluded from this study. Meanwhile, excluded subjects involved those with any history of known stroke (Hachinski ischemic score (HIS) ≥ 4), head injury, alcoholism, Parkinson's disease, epilepsy, major depression, other physical and mental illnesses, major medical illness (e.g., cancer, anemia and serious infection), and severe visual or hearing loss. All patients provided signed, informed consent to participate in the study, which was approved by the Research Ethics Committee of the Affiliated ZhongDa Hospital of Southeast University.

### Clinical data collection

General information, sociodemographic characteristics, as well as medical and psychological conditions of all participants were collected by a member of the research staff. Physical measurements, including weight, height, and blood pressure, were obtained using a standard beam balance scale. BMI was calculated as body weight divided by square of the height (kg/m^2^). Additional information, including age, gender, smoking history, diabetes duration, and insulin usage, were collected from available records. Glycemic and lipid profiles, including HbA1c (%), FBG, FIN, triglyceride, total cholesterol, high-density lipoprotein cholesterol and low-density lipoprotein cholesterol, were also collected in the clinic per the blood samples drawn from the subjects the following morning after their admission to the hospital. Insulin resistance was calculated according to the HOMA-IR formula: FBG (mmol/L) × FIN (mIU/L) / 22.5. Medical histories, which were self-reported or confirmed during their hospital stay, were also collected. Two blood pressure measurements ≥ 140/90 mmHg were considered as hypertension. Coronary artery disease diagnosis was documented on coronary angiography with ≥ 50% stenosis of a major epicardial coronary artery associated with positive stress test or ≥ 70% stenosis of a major epicardial coronary artery and classic angina [42]. DN is defined by increased urinary albumin excretion (UAE) (UAE > 20 μg/min) in the absence of other renal diseases [43]. Diabetic retinopathy is defined as the presence of typical retinal microvascular lesions in diabetic patients [44]. The preceding biochemical measurements of both groups were performed in the central laboratory of the Affiliated ZhongDa Hospital, which implements internal and external quality control procedures directed by the Chinese Laboratory Quality Control.

### Neuropsychological function evaluation

Participants completed neuropsychological testing comprising the following: overall cognitive function (MoCA), attention/psychomotor speed (DST, Trail Making Test A), visuospatial function (Clock Drawing Test), executive function (Word Similarity Test, Trail Making Test B), semantic memory (VFT) and episodic memory (AVLT). HIS, CDR, activity of daily living scale, and self-rating depression scale were also included. An experienced neuropsychiatry specialist facilitated the process for approximately 45 min by using a single-blind method.

### Blood sampling and plasma ghrelin measurements

Blood samples were simultaneously collected by venipuncture from MCI patients and controls between 6 and 7 a.m. following an overnight fast conducted the morning after their admission to the hospital. After centrifugation for 15 min, plasma was separated and stored at −80°C until analysis. We used a commercially available, sandwich enzyme-linked immunosorbent assay kit to measure plasma ghrelin concentrations per the manufacturer's instructions. Intra and interassay coefficients of variance were less than 10% and 12%, respectively. The sensitivity limit of the assay was 46.2 pg/mL.

### DNA extraction and single nucleotide polymorphism genotyping

Genomic DNA was extracted from the ethylenediaminetetraacetic acid-treated venous blood samples using a DNA purification kit (Puregene, Gentra Systems, Minneapolis, MN, USA). Polymerase chain reaction-restriction fragment length polymorphism was performed to detect variants of the ghrelin gene (rs4684677). The following forward and reverse primers were used: 5′-GAGGTGTCACTCAGCAGTCC-3′ and 5′-TCTTCTTCTTCAGGGCCTGGCTGTGCTGCT AGTAC-3′, respectively. PCR was conducted in a 10 μL reaction mixture containing 1 μL of genomic DNA, 1 U of Taq polymerase, 1.5 μL of 10 × PCR buffer, 1.2 μL of deoxynucleotide triphosphates, 0.9 μL of MgCl_2_, 0.75 μL of each primer and 5.325 μL of ddH2O. Amplification was initiated at 95°C for 5 min, followed by 45 cycles of denaturation at 95°C for 45 s, 60°C for 45 s, 72°C for 45 s and a final extension step at 72°C for 10 min. PCR products were digested using the specific restriction enzyme ScaI at 37°C for 3 h, followed by electrophoresis on a 3% agarose gel. The products were finally visualized under ultraviolet light after staining with ethidium bromide.

### Statistical analysis

Data are presented as *n* (%), mean ± standard deviation (SD), or median (interquartile range). Single sample Kolmogorov-Smirnov goodness-of-fit hypothesis test was used to show variable distribution. *P* > 0.05 was considered as normal distribution and vice versa. Sociodemographic, clinical characteristics and neuropsychological test scores of the MCI patients and control subjects were compared using Student's *t* test for normally distributed variables, and the nonparametric Mann-Whitney *U* test for asymmetrically distributed variables. Chi-square test was conducted to determine the distributions of categorical variables, and deviations from Hardy-Weinberg equilibrium (Santiago Rodriguez, Tom R. Gaunt, and Ian N. M. Day, Hardy-Weinberg Equilibrium Testing of Biological Ascertainment for Mendelian Randomization Studies). Partial correlation analysis was performed to explore the relationships between plasma ghrelin levels and cognitive performances, and binary logistic regression analysis was performed to estimate the predictor variables for MCI in T2DM patients. The cutoff value used in this study for suggested MCI was a MoCA score < 26, with a one-point adjustment of the total score for subjects with less than 12 years of education level. Statistical analyses were conducted using SPSS version 19.0 (SPSS Inc., Chicago, IL, USA). All tests were two-sided, and *p* < 0.05 was defined as statistically significant.
